# Newly learned shape–color associations show signatures of reliability-weighted averaging without forced fusion or a memory color effect

**DOI:** 10.1167/jov.22.13.8

**Published:** 2022-12-29

**Authors:** Stacey Aston, Cat Pattie, Rachael Graham, Heather Slater, Ulrik Beierholm, Marko Nardini

**Affiliations:** 1 Department of Psychology, Durham University, Durham, UK; 2 Biosciences Institute, Newcastle University, Newcastle, UK

**Keywords:** cue combination, novel cues, color perception, color memory

## Abstract

Reliability-weighted averaging of multiple perceptual estimates (or cues) can improve precision. Research suggests that newly learned statistical associations can be rapidly integrated in this way for efficient decision-making. Yet, it remains unclear if the integration of newly learned statistics into decision-making can directly influence perception, rather than taking place only at the decision stage. In two experiments, we implicitly taught observers novel associations between shape and color. Observers made color matches by adjusting the color of an oval to match a simultaneously presented reference. As the color of the oval changed across trials, so did its shape according to a novel mapping of axis ratio to color. Observers showed signatures of reliability-weighted averaging—a precision improvement in both experiments and reweighting of the newly learned shape cue with changes in uncertainty in Experiment 2. To ask whether this was accompanied by perceptual effects, Experiment 1 tested for forced fusion by measuring color discrimination thresholds with and without incongruent novel cues. Experiment 2 tested for a memory color effect, observers adjusting the color of ovals with different axis ratios until they appeared gray. There was no evidence for forced fusion and the opposite of a memory color effect. Overall, our results suggest that the ability to quickly learn novel cues and integrate them with familiar cues is not immediately (within the short duration of our experiments and in the domain of color and shape) accompanied by common perceptual effects.

## Introduction

Reliability-weighted averaging of multiple perceptual estimates (or cues) has been shown across a variety of perceptual domains for human observers (Landy, Banks, & Knill, 2011). For example, cues to depth are combined according to their reliabilities both across and within sensory modalities (Ernst & Banks, 2002; Hillis, Ernst, Banks, & Landy, 2002; Hillis, Watt, Landy, & Banks, 2004; Jacobs, 1999; Knill & Saunders, 2003; Nardini et al., 2010). Combining independent noisy estimates in this way is the optimal strategy for maximizing perceptual precision, as the resulting estimate is less variable (more reliable) than estimates from either single cue alone (Jones, 2016; Rohde et al., 2016).

Cue recruitment studies show that people can also learn to perceive via newly learned cues or statistical associations (Backus & Haijiang, 2007; Di Luca et al., 2010; Haijiang et al., 2006; Harrison & Backus, 2010; Harrison et al., 2011; Harrison & Backus, 2012) and recent cue combination studies show that newly learned cues can be combined with familiar cues to make more precise decisions (Aston, Beierholm, & Nardini, 2022; Ernst, 2007; Gibo, Mugge, & Abbink, 2017; Negen, Wen, Thaler, & Nardini, 2018, Negen, Bird, Slater, Thaler, & Nardini, 2021). For example, a newly learned audio cue to depth is combined with visual information to improve precision in depth judgements after short-term training (Negen et al., 2018, 2021). Similarly, along another dimension of spatial localization, novel visual cues are combined with familiar cues to increase the precision of horizontal location estimates (Aston et al., 2022). However, it remains unclear if novel cues have the same direct influence on perception as familiar cues or if they only influence final decision-making.

Here, we continue to expand on the limited work on efficient use of novel cues. In particular, we extend previous work on using new cues to localize objects in space (judging “where” they are) to a task in which new cues are used to match or recognize objects (judging “what” they are). We test participants in a short-term training study with a novel shape–color association, making object shape a useful cue to object color within the task. We test whether perceptual precision with the addition of this new cue, and further, we test whether any such abilities are accompanied by perceptual effects. To be clear, we define a perceptual effect as one where how the object appears to the participant changes based on the new cue, rather than the new cue only influencing final decision-making.

We introduce a novel shape–color association (a novel shape cue to color) by creating an arbitrary association between the axis ratio of an oval and its color. In each experiment, after a period of repeated exposure to the novel shape–color association, we tested for the two key signatures of reliability-weighted averaging that are commonly tested for in the cue combination literature: 1) a decrease in color matching variability when the shape cue is present and 2) reweighting of the shape cue with change in familiar cue uncertainty (Clark & Yuille, 1990; Holman, 1893; Jones, 2016; Rohde, van Dam, & Ernst, 2016). We also tested for a perceptual effect of the novel shape cue to color.

In summary, we found signatures of reliability-weighted averaging of the novel shape cue and familiar cues to color in both experiments. Participants showed a decrease in color matching variability in both experiments and cue reweighting with changes in uncertainty in Experiment 2. However, our results overall suggest that the ability to quickly learn novel cues and take a reliability-weighted average of novel and familiar cues is not immediately (within the short duration of our experiments) accompanied by common perceptual effects.

## Experiment 1

**Figure 1.**
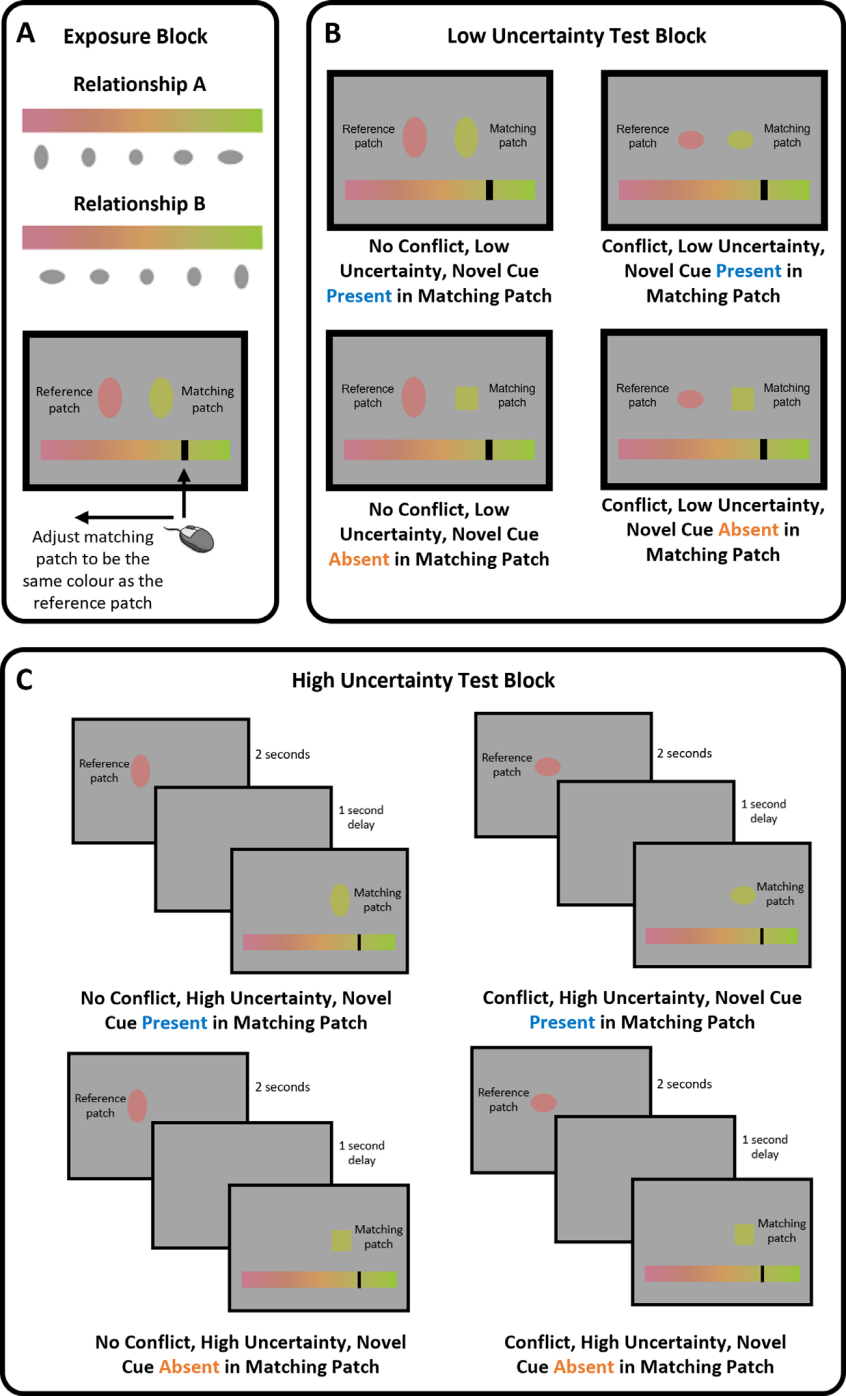
Adjustment trials in Experiment 1. (A) The experiment began with an exposure block where, through repeated simultaneous color matches, participants could learn the novel shape–color relationship. Participants learnt relationship A or B at random. (B) In the low uncertainty test block participants also completed simultaneous color matches, but the trials were now one of four types, varying whether the novel shape cue was present in the matching patch and whether the novel shape cue conflicted with the color of the reference patch according to the novel relationship introduced in the exposure block. (C) In the high uncertainty test block participants completed successive color matches, with the reference patch shown for 2 seconds and a delay of 1 second before presentation of the matching patch. Again, there were four types of trials in this block, varying novel cue presence in the matching patch and conflict between familiar and novel cues.

In Experiment 1, we introduce a novel shape cue to color by creating an arbitrary association between the axis ratio of an oval and its color (Figure 1). The direction of the association was varied across observers (tall oval mapping to a pinkish color and squashed to greenish or vice versa). For some participants, a taller oval mapped to a pinkish color and a wider oval mapped to a greenish color. For others, the mapping was reversed. We used both directions of mapping in order to avoid systematic effects of any preexisting associations between these cues.

To teach observers the novel relationship between shape and color, the experiment began with an exposure block, where participants were exposed to the novel relationship. On each trial in this block, participants were presented with a reference patch whose axis ratio was defined by the novel mapping from shape to color, and a matching patch with the same axis ratio as the reference patch. Participants were required to adjust the color of the matching patch to match that of the reference patch by moving a cursor controlled by the mouse along a color gradient. After the exposure block, we tested for 1) a decrease in matching variability when the novel cue was present in the matching patch compared with when it was absent (removing the novel cue by making the matching patch square), and 2) changing novel cue weights with changing familiar cue uncertainty. We increased familiar cue uncertainty by adding a delay between presentation of the reference and matching patches.

We tested for a perceptual effect of the novel shape cue to color in two ways. First, we included a simultaneous matching condition (the low uncertainty condition) where the novel cue was always present in the reference patch but could be present or absent in the matching patch. In this simultaneous matching condition, regardless of whether the novel cue is present or absent in the matching patch, the same information is available to the participants to complete the task (both familiar and novel cues to color are present on the screen). We propose that a reduction in matching variability when the novel shape cue is present in the matching patch (compared with when it is absent) in the simultaneous matching condition points toward a perceptual effect of the novel cue. The logic here is that if matching variability is decreased only when the novel cue is present in the matching patch, even though the same information is available in the reference patch when the novel cue is absent from the matching patch, then the novel shape cue has a perceptual effect on the appearance color of the matching patch, reducing appearance color variability when present and, subsequently, matching variability.

We also tested for a perceptual effect by testing for forced fusion. This phenomenon occurs when the perceptual system cannot help but take a reliability-weighted average of the available cues, even when keeping the cues separate and making a decision based on appearance due to each single cue alone would lead to better discrimination. Forced fusion has been documented in depth perception (Hillis et al., 2002; Nardini, Bedford, & Mareschal, 2010), visual–vestibular body rotation (Prsa, Gale, & Blanke, 2012), object recognition (Saarela & Landy, 2015), and also after learning to associate new arbitrary multisensory cues (Ernst, 2007), although that study did not also test for precision improvements via cue combination, our focus here.

Forced fusion is determined when an observer automatically fuses the available cues into a combined percept, even when doing so would make them worse at performing a particular task. Tasks used to test for this compare discrimination via a single cue with discrimination via a pair of conflicting cues. An observer who does not experience forced fusion can respond as precisely in the two-cue (conflicting) situation as in the single-cue condition, by attending to either single cue even in the two-cue (conflicting) case. In contrast, an observer who experiences forced fusion responds less precisely in the two-cue (conflicting) case because they average over the two conflicting cues. This averaging of opposing differences makes the stimuli being compared seem to be more similar; consequently, discrimination is less precise than when only one cue is available. We, therefore, consider fusion to be diagnostic of a potentially perceptual effect, where the observer comes to perceive the combined percept and not its constituent cues. Specially, we tested for forced fusion using a two-alternative forced choice (2AFC) task. The novel shape cue was either present (testing discrimination in a two-cue, conflicting situation) or absent (testing discrimination in a single-cue situation). In the task, one of the two alternatives was an exact match to the reference. The other alternative was offset from the reference in color and (when it was present) shape, but the offsets were opposing such that an equal weighting of familiar and novel cues would produce a matching color to the reference. Under the assumption of forced fusion, we expected thresholds to be higher when the novel cue was present, because conflicting information would make the two alternatives seem to be more similar.

We consider that a finding of forced fusion would point to a perceptual effect of the newly learned cue. However, because forced fusion is not found in all situations in which cues are combined to improve precision (Hillis et al., 2002), a negative finding would leave open the possibility that there are perceptual effects of the new cue, even if fusion is not one of them.

### Methods

#### Participants

We recruited 35 participants (30 female, age range 18–35 years) to take part in Experiment 1. One was excluded as they failed to meet our inclusion criteria (see Exclusion criteria), leaving 34 participants in the analysis. Participants were recruited through word of mouth, our department's undergraduate participant pool, and our laboratory’s Facebook page. Before they could take part in the experiment, participants were screened for color vision deficiencies using the Ishihara plates. All participants were compensated with £10 for their time plus an additional bonus of £5 to £10 depending on performance. If participants got *x*% of the total points available, they received £(*x* *+* 10)/10 bonus, rounded down, but received a minimum of £5.

#### Ethics

Ethical approval was received from the Durham University Psychology Department Ethics Board (reference number: 17/07). All participants gave written, informed consent prior to taking part in the experiment.

#### Apparatus

Stimuli were shown on a 10-bit ASUS Proart LCD screen with a width of 51 cm and a height of 29 cm (ASUS, Fremont, CA) in a dark room (no light source except the screen) with participants seated so that their eyes were approximately 60 cm from the screen. The monitor was controlled using a 64-bit Windows machine, equipped with an NVIDIA Quadro K600 10-bit graphics card (NVIDIA, Santa Clara, CA), running MATLAB scripts that used Psychtoolbox routines (Brainard, 1997; Kleiner, Brainard, & Pelli, 2007; Pelli, 1997). The stimuli were colorimetrically calibrated using a linearized calibration table based on measurements of the monitor primaries made with a Konica Minolta CS2000 spectroradiometer (Konica Minolta, Nieuwegein, the Netherlands). Conversions to CIELUV used the measured white point of the monitor: (*Y*, *x*, *y*) = (205.24,  0.31,  0.34) in CIE 1931 *Yxy* color space.

#### Stimuli

All colors used in the experiment were parameterized to fall along a gradient from pinkish to greenish. The gradient was defined as a chord of a hue circle (chroma = 85) in CIELUV chromaticity space. The start and end values of the chord had CIE 1931 chromaticities of (*x*, *y*) = (0.3386,  0.2821) and (*x*, *y*) = (0.3476,  0.3960) and a luminance of *Y* = 15 cd/m^2^. The color gradient was defined in this way to ensure perceptual uniformity. In other words, equal distances along the gradient should correspond with roughly equal perceptual differences. We arbitrarily assign a value of 0 to one end of the color gradient and 1 to the other. Then, all colors along the gradient can be specified by a number between 0 and 1. The end of the gradient that was assigned a value of 0 was decided at random for each participant.

The stimuli shown during the experiment were colored ovals (ellipses) in the colors described elsewhere in this article (Figure 1A). During the experiment, we created a novel association between the axis ratios (vertical/horizontal) of oval and their color. The axis ratio varied from 0.33 to 3.00, with the minimum and maximum axis lengths being 3% and 9% of the screen width, respectively. Importantly, as the axis ratio changed, the area of the oval was kept the same to ensure the colored patch had the same overall size in all case as this is known to affect color discrimination (e.g., Brown, 1952). The smallest and largest axis ratios were each identified with one of the arbitrary color values 0 or 1 across the color gradient. The smallest axis ratio was identified with either 0 or 1 at random for each participant. Thus, all colors could be identified with a unique axis ratio and, as the area was maintained, unique lengths for the vertical and horizontal axes. Each participant was exposed to one of the two possible mappings between color and shape, and we randomly determined which mapping each participant saw (Figure 1A).

#### Procedure

There were five blocks of trials in the task: the exposure block, the low uncertainty test block, the top-up exposure block, the high uncertainty test block, and the forced fusion block, each described in detail elsewhere in this article. Twenty-seven participants completed the blocks in the order described elsewhere in this article. We had intended to switch the ordering of the low and high uncertainty test blocks at random, but did not catch the error in the MATLAB code that meant all participants saw the same order until deep into data collection. At that point we edited the code and the last eight participants recruited completed the blocks in the switched order. All participants completed testing within a single session, typically lasting between 1.5 and 2.0 hours.

The exposure block, top-up block, and test blocks consisted of adjustment trials, where participants could adjust the color of one patch (the matching patch) to match the color of another patch (the reference patch). There were eight types of adjustment trials as the shape of the matching patch, the delay between the reference and matching patch, and whether the familiar and novel cues were in conflict could vary. These were important manipulations for addressing our research questions. The shape of the reference patch (oval or square) manipulated the presence of the novel shape cue in the matching patch (novel cue present or absent). The delay between presentation of the reference and matching patch (no delay or 1 second delay) manipulated the uncertainty of familiar (low-level) cues to color (low or high familiar cue uncertainty). Adding conflict between the familiar cues to color and novel shape cue to color allowed us to measure the amount of weight that participants placed on the novel cue.

During the exposure block (Figure 1A), adjustment trials were all the same type: no conflict, low uncertainty trials with the novel cue present in the matching patch. Participants were simultaneously presented with the reference patch (on the left of the screen) and the matching patch (on the right of the screen). The color of the reference patch had a value between 0.05 and 0.95 on our 0 to 1 reference scale (described elsewhere in this article) in steps of 0.05, each shown 10 times over 190 trials in total. Both the reference and matching patch were ovals with the axis ratio that corresponded with the color value of the reference patch. The starting color of the matching patch was a random value between 0 and 1. At the bottom of the screen, participants saw the whole color gradient as a horizontal bar. By moving the mouse, they could move a black vertical line left and right across the gradient, changing the color of the matching patch, and left click to issue a response. After responding, participants received feedback gaining a number of points for that trial based on performance. Points were awarded according to a mean squared error loss function. The ideal response for the loss function was set as the average of the color of the reference patch and the color that the shape of the reference patch corresponded with. Participants were only shown their score as feedback and did not receive information regarding the offset of their response from the ideal value or any information regarding the ideal value itself. We gave feedback in this way to avoid a situation where participants responses were biased toward the value suggested by the novel shape cue, but only because they had learned to adjust their responses according the trial-by-trial feedback of their error. We chose not to use the color of the reference patch alone as the ideal value to encourage participants to explore the benefit they could gain from using the shape cue.

Adjustment trials in the low uncertainty test block were the same as in the exposure block, except that the matching patch could be a square on some trials and/or the axis ratio of the oval could conflict with the corresponding color (Figure 1B). Thus, there were four types of trials in this block: 1) no conflict, low uncertainty trials with the novel cue present in the matching patch; 2) no conflict, low uncertainty trials with the novel cue absent in the matching patch; 3) conflicting, low uncertainty trials with the novel cue present in the matching patch; and 4) conflicting, low uncertainty trials with the novel cue absent in the matching patch. For trials where there was no conflict, regardless of whether the novel cue was present or absent in the matching patch, the color of the reference patch had an arbitrary value between 0.05 and 0.95 in steps of 0.10, each repeated five times for each trial type. In trials without conflict and where the novel cue was present in the matching patch, the reference and matching shape were ovals with axis ratios corresponding with the color of the reference patch. In trials without conflict and where the novel cue was absent from the matching patch, the matching patch was a square with side length 5.2% of the screen width. For the trials with conflict, regardless of whether the novel cue was present or absent in the matching patch, the color of the reference patch had an arbitrary value of 0.25 or 0.75, but the axis ratio of the reference oval conflicted with this value by –10%, –5%, 5%, or 10% of the mapping. These conflict values were chosen in early piloting with members of the laboratory. Through trial and error, we chose these values to ensure a conflict large enough to affect matches, but not so big that the conflict was visible to the participant. In trials with conflict and where the novel cue was present in the matching patch, the matching patch was also an oval with the conflicting value. In trials with conflict and where the novel cue was absent from the matching patch, the matching patch was a square with side length 5.2% of the screen width. There were 5 repeats of each reference and conflict value pairing for each trial type and a total of 180 trials in this block in total.

The top-up exposure block was identical to the exposure block (Figure 1A), containing only no conflict, low uncertainty trials with the novel cue present in the matching patch, except that the number of repeats for each reference was decreased to 3 for 57 trials in this block in total.

The high uncertainty test block was identical to the low uncertainty test block except that the reference patch was now only shown for 2 seconds, and participants were only allowed to issue their response after a 1-second delay (Figure 1C). Hence, this block also contained four types of trials: 1) no conflict, high uncertainty trials with the novel cue present in the matching patch; 2) no conflict, high uncertainty trials with the novel cue absent in the matching patch; 3) conflicting, high uncertainty trials with the novel cue present in the matching patch; and 4) conflicting, high uncertainty trials with the novel cue absent in the matching patch.

**Figure 2.**
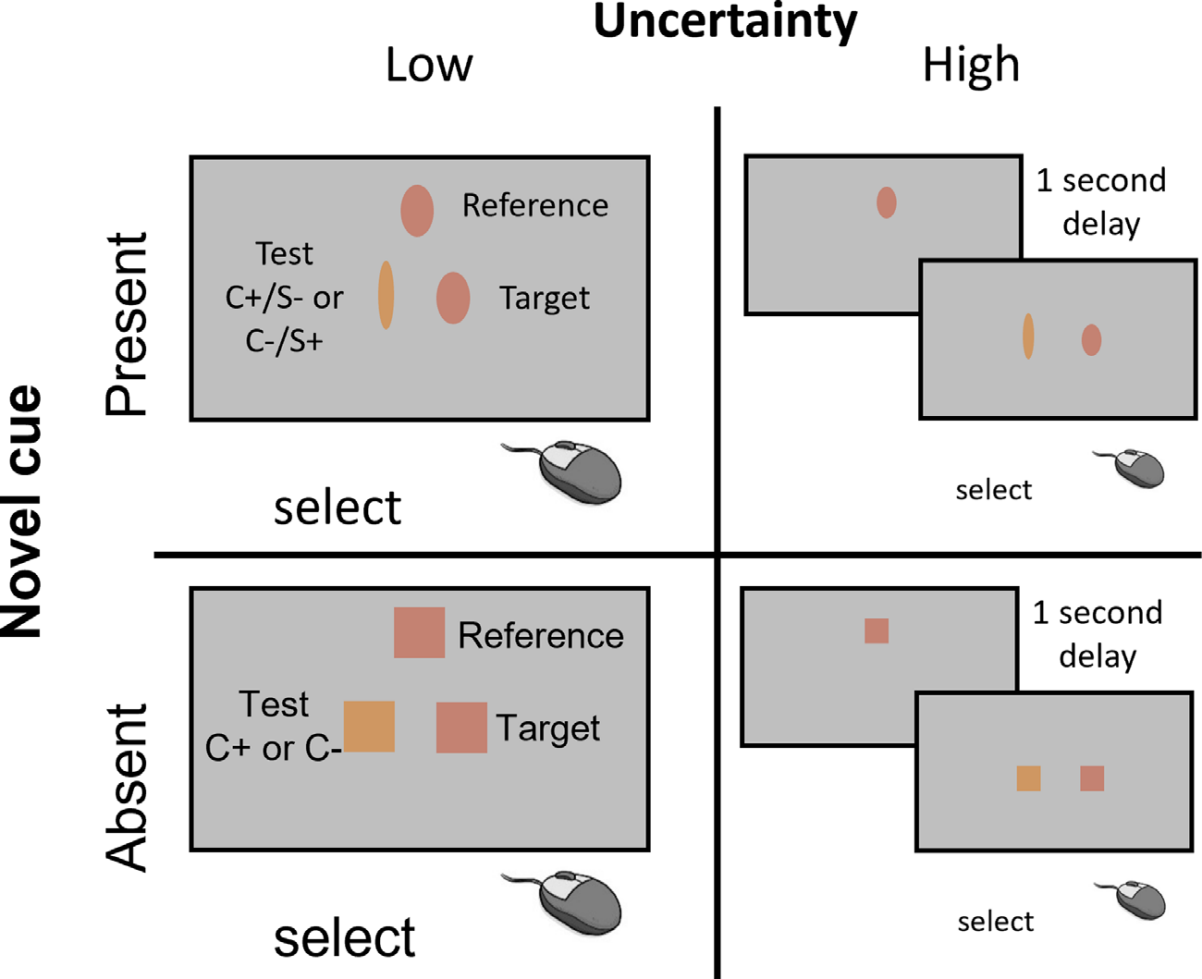
Forced fusion trials in Experiment 1. This part of the experiment consisted of two-alternative forced-choice trials that could be one of four types. The four trial types were defined by two varying factors: novel cue presence and familiar cue uncertainty. We varied novel cue presence by making reference and comparisons (the test and target patches) ovals, with axis ratios defined by the novel shape–color relationship (novel cue present) or squares (novel cue absent). We varied familiar cue uncertainty by either presenting the reference and comparison patches simultaneously or adding a 1-second delay between the presentation of the reference patch and the comparisons.

The forced fusion block (Figure 2) was designed to test for forced fusion of the newly learned novel shape cue and familiar cues to color. To test for forced fusion in this block participants completed a 2AFC task. In the task, one of the two alternatives was an exact match to the reference. The other alternative was offset from the reference in both shape and color, but the offsets were opposing such that an equal weighting of familiar and novel cues would produce a matching color to the reference. We also manipulated whether the novel cue was present or absent to establish baseline thresholds for discrimination. Under the assumption of forced fusion, we expected thresholds to be higher when the novel cue was present because conflicting information should make the two alternatives appear to be more similar.

Trials in the forced fusion block were split into two sections, one containing low uncertainty 2AFC trials and the other containing high uncertainty 2AFC trials. Whether the low or high uncertainty trials came first was randomized for each participant.

In the low uncertainty 2AFC trials, participants were presented with a reference patch of color at the top of the screen and two comparisons below it. In some trials, the novel shape cue was present (novel present, low uncertainty 2AFC trials) and in others it was absent (novel absent, low uncertainty 2AFC trials). In novel absent, low uncertainty 2AFC trials, the reference and comparison patches of color were all squares with side length 5.2% of the screen width. One of the comparisons was a perfect color match to the reference (the target), the other varied from the reference by a different amount on each trial (the test), determined by a 1-up, 1-down staircase procedure with a step size of 0.02 that terminated after 35 trials or 15 reversals. The staircase procedure was used to establish 50% discrimination thresholds around each of two reference values, namely, 0.25 and 0.75. One staircase approached each reference from either direction with starting differences between the reference and test patches of –0.1 or 0.1. Thus, there were four novel absent, low uncertainty staircases. These staircases established baseline 50% color discrimination thresholds for each reference and in each direction along the color gradient.

There were also four novel present, low uncertainty staircases made up of novel present, low uncertainty 2AFC trials. These trials/staircases were identical to the novel absent, low uncertainty trials/staircases, except that all patches were ovals. The reference and target patches had axis ratios determined by the novel mapping from their color to oval axis ratio, but the axis ratio of the test patch conflicted with its color. If the difference between the color of the reference and test patch was X, the difference between the axis ratio of the reference and the test patch was –X. Subsequently, if the color and shape cues were fused, thresholds should be larger when the novel cue is present as the test and match patches should appear more similar. All novel absent and novel present, low uncertainty staircases were interleaved so that there were eight staircases running simultaneously in this section of the forced fusion block.

There was a corresponding high uncertainty 2AFC trial or staircase for each type of low uncertainty 2AFC trial or staircase. The only difference between low and high uncertainty 2AFC trials was that in the high uncertainty 2AFC trials, the reference patch was shown alone for two seconds and then there was a 1-second delay (where a blank gray screen was shown) before presentation of the two comparisons. The eight high uncertainty staircases were interleaved in a different section of the forced fusion block.

We only report data from the forced fusion block for 30 of the 35 participants. After the first four participants, we refined the code so that it did not allow for the sign of conflicts to change, effectively preventing staircases from crossing over, something we did not consider until we saw it happen in a dataset. One further participant was excluded after reporting to the experimenter that they were trying to pick the comparison that did not match the reference rather than the one that did.

#### Data analysis

All calculations were performed in terms of screen proportions, and we calculated three different types of measures in this experiment. To test for signatures of reliability-weighted averaging we calculated matching variability (variable error) and the weight placed on the novel cue from different types of adjustment trials in blocks 2 and 4. To test for a perceptual effect of the novel cue, we calculated discrimination thresholds from the staircases in the forced fusion block.

Matching variability was calculated for each type of no conflict adjustment trial in blocks 2 and 4: no conflict, low uncertainty trials with the novel cue present in the matching patch or the novel cue absent in the matching patch (block 2) and no conflict, high uncertainty trials with the novel cue present in the matching patch or the novel cue absent in the matching patch (block 4). Thus, we calculated matching variability for four types of adjustment trials.

To calculate matching variability we used a method described fully elsewhere (Aston, Negen, Nardini, & Beierholm, 2021). In brief, the method is designed to account for central biases in continuous responses that may decrease statistical power for detecting a gain in precision using multiple cues. To calculate measures of variability according to the method, we regress matches for each trial type on the true color value and calculate the standard deviation of the residuals. If the slope of the fitted regression line is significantly less than one, the standard deviation of the residuals is divided by the fitted slope of the regression line to correct for a central bias. If there is no evidence of a central bias (the slope is not significantly less than one), no correction is performed.

The weight placed on the novel cue was calculated for each of the four types of conflict adjustment trials in blocks 2 and 4: 1) conflicting, low uncertainty trials with the novel cue present in the matching patch (block 2); 2) conflicting, low uncertainty trials with the novel cue absent in the matching patch (block 2); 3) conflicting, high uncertainty trials with the novel cue present in the matching patch (block 4); and 4) conflicting, high uncertainty trials with the novel cue absent in the matching patch (block 4).

To calculate the weight placed on the novel cue we again used a method that is described fully elsewhere (Aston et al., 2021). In brief, we model matches, *m*, as

$$m=\left(1-w_{c}\right)\times \left[w_{s}s+\left(1-w_{s}\right)f\right]+w_{c}\times \;0.5+\varepsilon ,$$

where *s* is the value of the shape cue, *f* is the value of low-level familiar cues to color, *w_s_* is the weight on the shape cue, *w_c_* is the strength of the central bias (the central value being 0.5), and $\varepsilon ∼N(0,\sigma_{n}^{2})$ is an additional noise term. We estimate the parameters *w_s_*, *w_c_*, and σ_*n*_ using a Gibbs Sampler (JAGS; Plummer, 2003) implemented in MATLAB using the MATLAB-to-JAGS interface. We ran three independent chains, discarding the first 100 samples of each chain as burn-in, and recording 1000 samples after the burn-in period, thinned by recording only every fifth sample. Both fitted weights (*w_s_* and *w_c_*) were initialized at 0.5 in all chains. The standard deviation of the additional noise (σ_*n*_) was initialized at 0.01. The resulting estimates were taken as the mean of the expected values from the three chains.

To calculate thresholds from the staircases in the forced fusion block we took the average of the last two reversals from each staircase.

#### Exclusion criteria

We planned to exclude participants from the analyses if their matching variability was too high for either of the low or high uncertainty conditions. Match variability had to be less than 0.075 for the low uncertainty condition and less than 0.150 for the high uncertainty condition. We chose these values so that match variability would not be so high in the high uncertainty condition that participants would struggle with the task and become uninterested when they failed to score many points. We set the low uncertainty threshold at one-half of the high uncertainty threshold to ensure a good difference between variability in the two conditions. The one participant that was excluded from Experiment 1 was excluded because they did not meet the inclusion criteria in the high uncertainty condition when the novel cue was absent.

### Results

#### Matching variability decreased when the novel shape cue was present in the matching patch, but only in the low uncertainty condition

**Figure 3.**
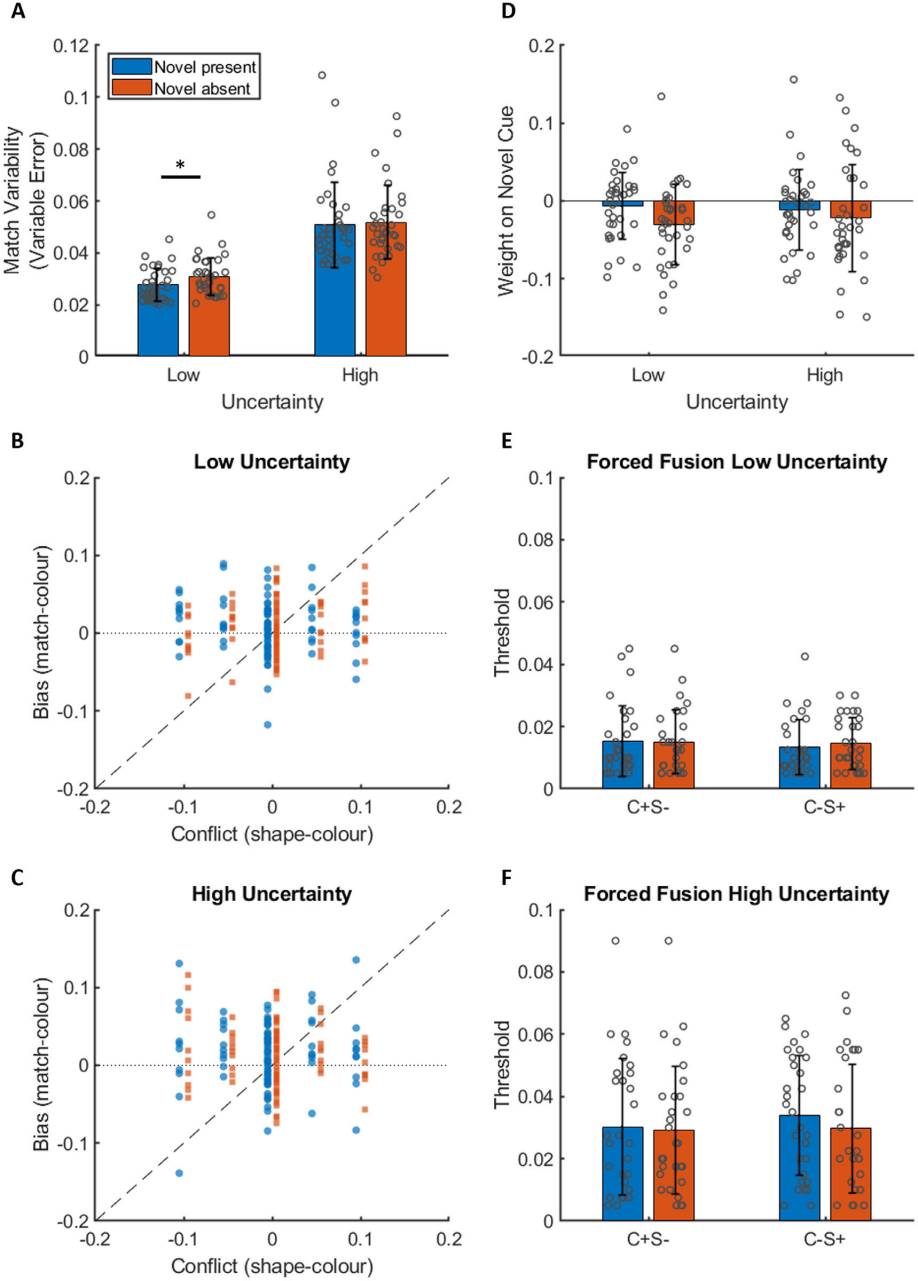
Results from Experiment 1. (A) Matching variability (variable error) for each level of uncertainty split by novel cue presence. (B and C) Individual matching biases (matched color – reference color) for an example participant plotted against the conflict between the shape (novel) and color (familiar) cues in the reference patch (shape cue value – color cue value) in the low (B) and high (C) uncertainty conditions. Blue dots are when the novel cue was present in the matching patch. Orange squares are when the novel cue was absent from the matching patch. (D) The inferred weight on the novel cue in each condition. (E and F) Discrimination thresholds from the forced fusion task for each condition. Error bars are ±1 standard deviation.

Figure 3A shows matching variability (variable error) in both the low (simultaneous matching) and high (successive matching) uncertainty conditions when the novel shape cue was present in the matching patch and when the novel shape cue was absent from the matching patch. Under the assumption of reliability-weighted averaging, we expected matching variability to be lower when the novel shape was present in the matching patch. In separate Wilcoxon signed-rank tests, we found significantly lower matching variability when the novel cue was present in the low uncertainty condition, *z* =   −3.7,  *p* < 0.001, but no difference between when the novel cue was present and when it was absent in the high uncertainty condition, *z* =   −0.44,  *p* =  0.663.

#### When novel and familiar cues conflicted, the weight on the novel cue did not differ significantly from zero, regardless of novel cue presence in the matching patch or level of uncertainty

Figures 3B and 3C show data from an example participant on trials where the novel shape cue conflicted with familiar cues to color. In these figures we plot the conflict (shape value – true color value) on the horizontal axis and response bias (match value – true color value) on the vertical axis. Blue dots are when the novel cue was present in the matching patch. Orange squares are when the novel cue was absent from the matching patch. Under the assumption of reliability-weighted averaging, conflicting novel shape cues should bias matches away from the true color value and toward the conflicting novel shape cue value. These figures suggest this was not the case for this participant, who placed approximately zero weight on the novel shape cue regardless of whether the novel shape cue was present or absent in the matching patch. Indeed, when we fit the model described in the data analysis section to each participant's data individually, we find that the fitted weights on the novel shape cue, *w_s_*, are not significantly greater than zero in either uncertainty condition, regardless of whether the novel cue was present or absent (novel absent, low uncertainty: $\bar{x}\;=\;-0.03,\;t\left(33\right)\;=\;-3.42,\;p\;=\;0.999$; novel absent, high uncertainty: $\bar{x}\;=\;-0.02,\;t\left(33\right)\;=\;-1.84,\;p\;=\;0.962$; novel present, low uncertainty: $\bar{x}\;=\;-0.01,\;t\left(33\right)\;=\;-0.92,\;p\;=\;0.817$; novel present, high uncertainty: $\bar{x}\;=\;-0.01,\;t\left(33\right)\;=\;-1.25,\;p\;=\;0.89$ (Figure 3D).

#### There was no evidence of forced fusion, regardless of novel cue presence in the matching patch or level of uncertainty

If the novel shape cue was subject to forced fusion with familiar shape cues to color, then we expect thresholds in the forced fusion block to be higher when the novel shape cue is present, because the conflicting shape information would make the test patch look more like the target patch than when the judgement is made based on familiar cues alone (when the novel shape cue is absent). Conversely, if novel and familiar cues are not subject to forced fusion the conflicting shape information can be ignored, and thresholds should be the same regardless of novel cue presence in all conditions. Figures 3E and 3F suggest that thresholds were not increased when the novel cue was present regardless of conflict direction and regardless of level of uncertainty. Indeed, a 2 (conflict direction: C+S– or C–S+) × 2 (novel cue presence: present or absent) × 2 (uncertainty: low or high) repeated measures analysis of variance finds no three-way interaction effect, *F*(1, 28)  =  0.76,  *p*  =  0.340, no two-way interaction effect of cue presence and either of the other factors, cue presence × conflict direction: *F*(1, 28)  = 0.14,  *p*  = 0.713; cue presence × uncertainty: *F*(1, 28)  =  2.15,  *p*  =  0.015), and no main effect of cue presence, *F*(1, 28)  =  0.80,  *p*  =  0.378). There was a main effect of uncertainty with significantly higher thresholds in the high uncertainty condition, *F*(1, 28)  =  35.98,  *p*  <  0.001.

## Interim discussion

In Experiment 1, we found weak evidence for reliability-weighted averaging. In the low uncertainty condition, variability was decrease when the novel cue was present in the matching patch, suggesting that participants could combine the novel and familiar cues to enhance perception. However, there was no decrease in variable error when the novel cue was present in the matching patch in the high uncertainty condition and the inferred weight placed on the novel cue was not significantly different to zero in any condition.

We also found mixed evidence for a perceptual effect of the novel cue. In the low uncertainty condition, where participants made simultaneous matches and all information (novel and familiar cues) was present on the screen regardless of whether the novel cue was present in the matching patch, matching variability was lower when the novel cue was present in the matching patch. Recall that, in this simultaneous matching condition, regardless of whether the novel cue is present or absent in the matching patch, the same information is available to the participants to complete the task (both familiar and novel cues to color are present on the screen). Thus, we propose that this result points toward a perceptual effect of the novel cue, because there was a decrease in matching variability when the novel shape cue was present in the matching patch but not when it was absent, even though the same information is available in the reference patch when the novel cue is absent from the matching patch. However, we did not find any evidence of a perceptual effect in the forced fusion task.

It is possible that we only saw a decrease in variability in the low and not high uncertainty condition because the effect is small and data in the high uncertainty condition are noisier than in the low uncertainty condition, making the effect more difficult to detect. Similarly, the fact that weight on the novel cue was not significantly different to zero when cues were conflicting suggests that the reliabilities of the two cues are mismatched, with the familiar cue being much more reliable than the novel shape cue. Thus, it is not surprising that we found no evidence of forced fusion, because little weight would be placed on the conflicting cue. These initial results are therefore somewhat inconclusive, possibly with reasons to do with the reliabilities of the two cues. We addressed these issues in Experiment 2.

## Experiment 2

In Experiment 2, we decreased the reliability of the familiar cue to better match the reliability of the novel cue and encourage participants to place more weight on the novel cue. We decreased familiar cue reliability by adding chromatic noise to the reference patch and having a delay between the presentation of the reference and matching patch in both high and low uncertainty conditions. We again tested for a perceptual effect of the novel cue, but in a different way that did not require cue reliabilities to be well matched. Here, we tested for a perceptual effect by testing for a memory color effect (Witzel & Hansen, 2015), such as when a gray banana appears slightly yellow (Hansen, Olkkonen, Walter, & Gegenfurtner, 2006; Olkkonen, Hansen, & Gegenfurtner, 2008; Witzel, Valkova, Hansen, & Gegenfurtner, 2011). Hansen et al. (2006) illustrated this effect by asking participants to adjust the color inside the outline of a banana until it appeared gray, showing that the gray match made inside the banana shape was bluer than a control match to gray. This finding suggests that participants had to compensate for the banana appearing yellow (the memory color effect) by adding blue to the match. This literature suggests that this is a promising domain in which to look for perceptual effects of a newly learned novel shape cue to color as the familiar shape of an object has been shown to affect color appearance.

### Methods

#### Participants

We recruited 45 participants (30 female; age range, 18–32 years) to take part in Experiment 2. Fifteen participants were excluded because they failed to meet our inclusion criteria (as delineated elsewhere in this article). Participants were recruited through word of mouth, our department's undergraduate participant pool, and our laboratory’s Facebook page. Before they could take part in the experiment, participants were screened for color vision deficiencies using the Ishihara plates. All participants were compensated with £10 for their time plus an additional bonus of £5 to £10 depending on performance. If participants got *x*% of the total points available, they received £(*x* + 10)/10 bonus, rounded down, but received a minimum of £5.

#### Ethics

Ethical approval was received from the Durham University Psychology Department Ethics Board (reference number: 17/07). All participants gave written, informed consent prior to taking part in the experiment.

#### Apparatus

We used the same apparatus as that used for Experiment 1.

#### Stimuli

**Figure 4.**
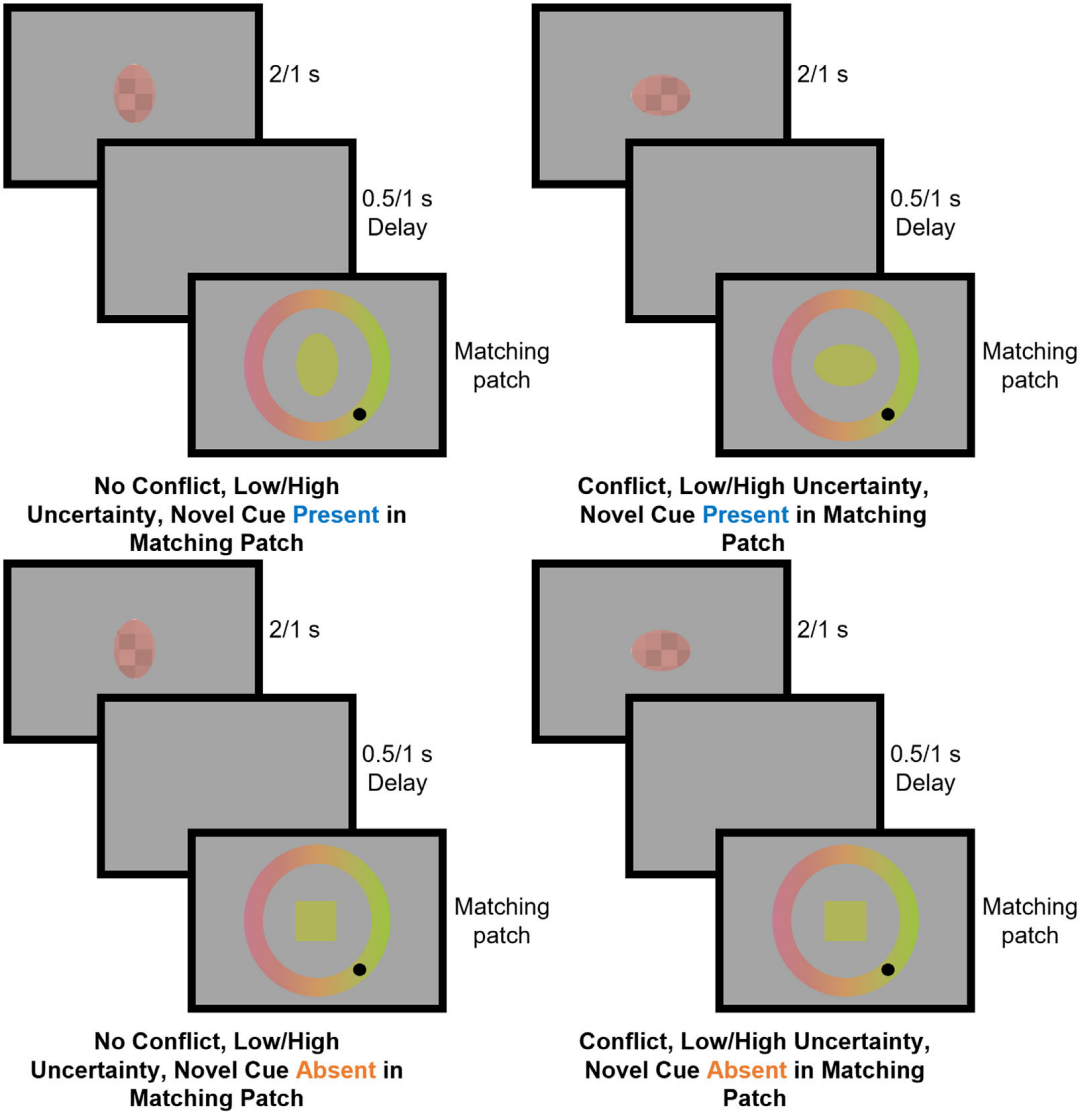
Adjustment trials in the test blocks of Experiment 2. There were two blocks of test trials in Experiment 2, one with low uncertainty (a reference patch presentation time of 2 seconds and a delay of 0.5 seconds before presentation of the matching patch) and one with high uncertainty (a reference patch presentation time of 1 second and a delay of 1 second before presentation of the matching patch). In each test block, trials were one of four types, varying whether the novel shape cue was present in the matching patch and whether the novel shape cue conflicted with the color of the reference patch according to the novel relationship introduced in a preceding exposure block.

We used the same gradient of colors and the same mapping between colors and axis ratios as in Experiment 1. To decrease familiar cue reliability, we added chromatic noise to the reference stimulus (Figure 4). To add chromatic noise, reference patches were filled with a checkerboard pattern with each check on the board filled with either the true reference color, or the true reference color plus or minus a small jitter. The jitter was 0.03 of the color gradient in the low chromatic noise condition and 0.1 of the color gradient in the high chromatic noise condition. How the checkerboard was filled was pseudo-randomized to ensure that the mean color was with 1.5 Δ*E* of the true reference color in CIELUV.

#### Procedure

There were four main blocks of trials in the task: the exposure block, the low uncertainty test block, the top-up exposure block, and the high uncertainty test block. The order of the low and high uncertainty test blocks was switched at random for each participant. Interleaved with the four main blocks of trials were sets of achromatic matches. There was a control set of achromatic matches at the start of the experiment and a test set of achromatic matches at the end of each block of trials resulting in five sets of achromatic matches in total.

The exposure, top-up, and low or high uncertainty test blocks consisted of adjustment trials, where participants could adjust the color of one patch (the matching patch) to match the color of another patch (the reference patch). There were eight types of adjustment trials because the shape of the matching patch, the noise in the reference patch, and delay between the reference and matching patch, and whether the familiar and novel cues were in conflict could vary. As in Experiment 1, these manipulations addressed our research questions. The shape of the reference patch (oval or square) manipulated the presence of the novel shape cue in the matching patch (novel cue present or absent). The noise in the reference patch, duration of the reference patch, and delay between presentation of the reference and matching patch (low noise/2-second duration/500-millisecond delay or high noise/1-second duration/1-second delay) manipulated the uncertainty of familiar (low-level) cues to color (low or high familiar cue uncertainty). Adding conflict between the familiar cues to color and novel shape cue to color allowed us to measure the amount of weight that participants placed on the novel cue.

During the exposure block adjustment trials were all the same type: no conflict, low uncertainty trials with the novel cue present in the matching patch. Participants were first presented with the reference patch for 2 seconds and the matching patch 500 milliseconds later (Figure 4). The mean color of the reference patch had a value between 0.10 and 0.90 in steps of 0.05, each shown 11 times for 187 trials in total. We had to decrease the range of stimuli shown in Experiment 2 (compared with Experiment 1) to allow for variation of the color in the reference patch (the addition of chromatic noise). Both the reference and matching patch were ovals with the axis ratio that corresponded with the color value of the reference patch. The starting color of the matching patch was a random value between 0 and 1. Around the matching patch, participants saw the whole color gradient as a ring. By moving the mouse, they could move a black circle around the ring, changing the color of the matching patch, and left click to issue a response. After responding, participants received feedback in the form of points for that trial. Points were awarded according to a mean squared error loss function.

Adjustment trials in the low uncertainty test block were the same as in the exposure block, except that the matching patch could be a square on some trials and/or the axis ratio of the oval could conflict with the corresponding color (Figure 4). Thus, there were four types of trials in this block: 1) no conflict, low uncertainty trials with the novel cue present in the matching patch; 2) no conflict, low uncertainty trials with the novel cue absent in the matching patch; 3) conflicting, low uncertainty trials with the novel cue present in the matching patch; and 4) conflicting, low uncertainty trials with the novel cue absent in the matching patch. For trials where there was no conflict, regardless of whether the novel cue was present or absent in the matching patch, the mean color of the reference patch had an arbitrary value between 0.1 and 0.9 in steps of 0.1, each repeated five times for each trial type. In trials without conflict and where the novel cue was present in the matching patch, the reference and matching shape were ovals with axis ratios corresponding with the color of the reference patch. In trials without conflict and where the novel cue was absent from the matching patch, the matching patch was a square with side length that was 5.2% of the screen width. For the trials with conflict, regardless of whether the novel cue was present or absent in the matching patch, the mean color of the reference patch had an arbitrary value of 0.25 or 0.75, but the axis ratio of the reference oval conflicted with this value by –10%, –5%, 5%, or 10% of the mapping. In trials with conflict and where the novel cue was present in the matching patch, the matching patch was also an oval with the conflicting value. In trials with conflict and where the novel cue was absent from the matching patch, the matching patch was a square with side length 5.2% of the screen width. There were five repeats of each reference and conflict value pairing for each trial type and a total of 170 trials in this block in total.

The top-up exposure block was identical to the exposure block, containing only no conflict, low uncertainty trials with the novel cue present in the matching patch, except that the number of repeats for each reference was decreased to 4 for 68 trials in this block in total.

The high uncertainty test block was identical to the low uncertainty test block, except that the reference patch was now only shown for 1 second, and participants were only allowed to issue their response after a 1-second delay (Figure 4). Hence, this block also contained four types of trials: 1) no conflict, high uncertainty trials with the novel cue present in the matching patch; 2) no conflict, high uncertainty trials with the novel cue absent in the matching patch; 3) conflicting, high uncertainty trials with the novel cue present in the matching patch; and 4) conflicting, high uncertainty trials with the novel cue absent in the matching patch.

The control set of achromatic matches was designed to measure a control match to gray for each participant. This set of matches was at the start of the experiment before any exposure to the novel shape–color association. Participants made five matches to gray with the shape varying (arbitrary values of 0.1, 0.3, 0.5, 0.7, and 0.9) and the order of the shapes randomized. Participants were instructed to “adjust the patch so that it appears gray (neither blue, yellow, red, or green).” They made the adjustment using the left, right, down, and up arrow keys on the keyboard. Each key changed the color by 0.25 Δ*E* in CIELUV color space. The up key increased the value along the v* axis, the down key decreased the value along the v* axis, the right key increased the value along the u* axis, and the left key decreased the value along the u* axis.

In each subsequent set of achromatic matches, participants did the same as for the control set of achromatic matches, except that there were three repeats of each shape in a random order.

#### Data analysis

**Figure 5.**
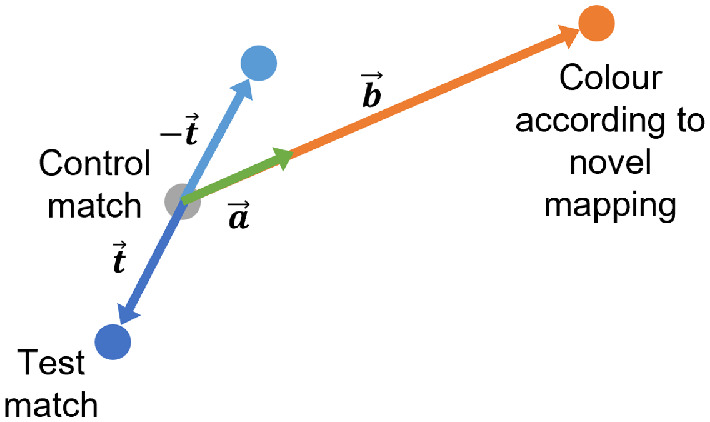
Calculating memory color indices. Illustration within a schematic two-dimensional color space. The gray dot represents the control match. The orange dot represents the color of the shape being adjusted according to the novel mapping between shape and color. $\vec{b}$ is the vector from the control match to the color according to the novel mapping. The dark blue dot represents the test match made by the participant. $\vec{t}$ is the vector from the control match to the test match, which we expect to go in the opposite direction to $\vec{b}$ under the assumption of a memory color effect. Thus, to quantify the effect we project $-\vec{t}$ onto $\vec{b}$ to find $\vec{a}$. The size of the memory color effect can then be calculated as the magnitude of $\vec{a}$ over the magnitude of $\vec{b}$.

Data from the adjustment trials was analysed in the same way as in Experiment 1. To analyze the achromatic matches we calculated memory color indices (Figure 5). Memory color indices were calculated separately for each test shape and each set of achromatic matches. For test shape X in achromatic match set Y, we calculated the memory color index as

$$\begin{array}{c} m=\frac{\left|\vec{a}\right|}{\left|\vec{b}\right|}=\;\frac{\vec{b}\cdot -\vec{t}}{{\left|\vec{b}\right|}^{2}} \end{array}$$

where $\vec{t}$ is the vector between the mean control match (the mean over the five matches in the control set of achromatic matches) and the mean shape X match in set Y, $\vec{b}$ is the vector between the mean shape X match in set Y and the color of the shape according to the novel mapping, and $\vec{a}$ is the vector projection of $-\vec{t}$ on $\vec{b}$. Subsequently, a positive color memory index indicates the presence of the classic color memory effect, where achromatic matches are pushed away from the associated shape color toward the opposing hue. A larger positive color memory index indicates a larger memory color effect. A negative color memory index indicates the opposite of the classic color memory effect, where achromatic matches are pulled toward the associated shape color. A smaller negative color memory index indicates a larger opposite effect.

#### Exclusion criteria

As in Experiment 1, we planned to exclude participants from the analyses if their matching variability was too high for either of the low or high uncertainty conditions. Matching variability had to be less than 0.075 for the low uncertainty condition and less than 0.150 for the high uncertainty condition. Of the 15 participants excluded in Experiment 2, two were exclude because they did not meet the inclusion criteria in the low uncertainty condition when the novel cue was absent, three were excluded because they did not meet the inclusion criteria in the low uncertainty condition when the novel cue was present, one was excluded because they did not meet the inclusion criteria in the high uncertainty condition when the novel cue was present, and others were excluded because they failed to meet the inclusion criteria in multiple conditions; for example, two failed to meet the inclusion criteria for both low uncertainty conditions.

### Results

#### Matching variability decreased when the novel shape cue was present in the matching patch, but only in the low uncertainty condition

As in Experiment 1, we found significantly lower matching variability when the novel cue was present in the low uncertainty condition, *z*  =   −2.07,  *p*  = 0.039, but no difference between when the novel cue was present and when it was absent in the high uncertainty condition, *z*  =  0.38,  *p*  = 0.704).

#### When novel and familiar cues conflicted, the weight placed on novel cue was flexible

**Figure 6.**
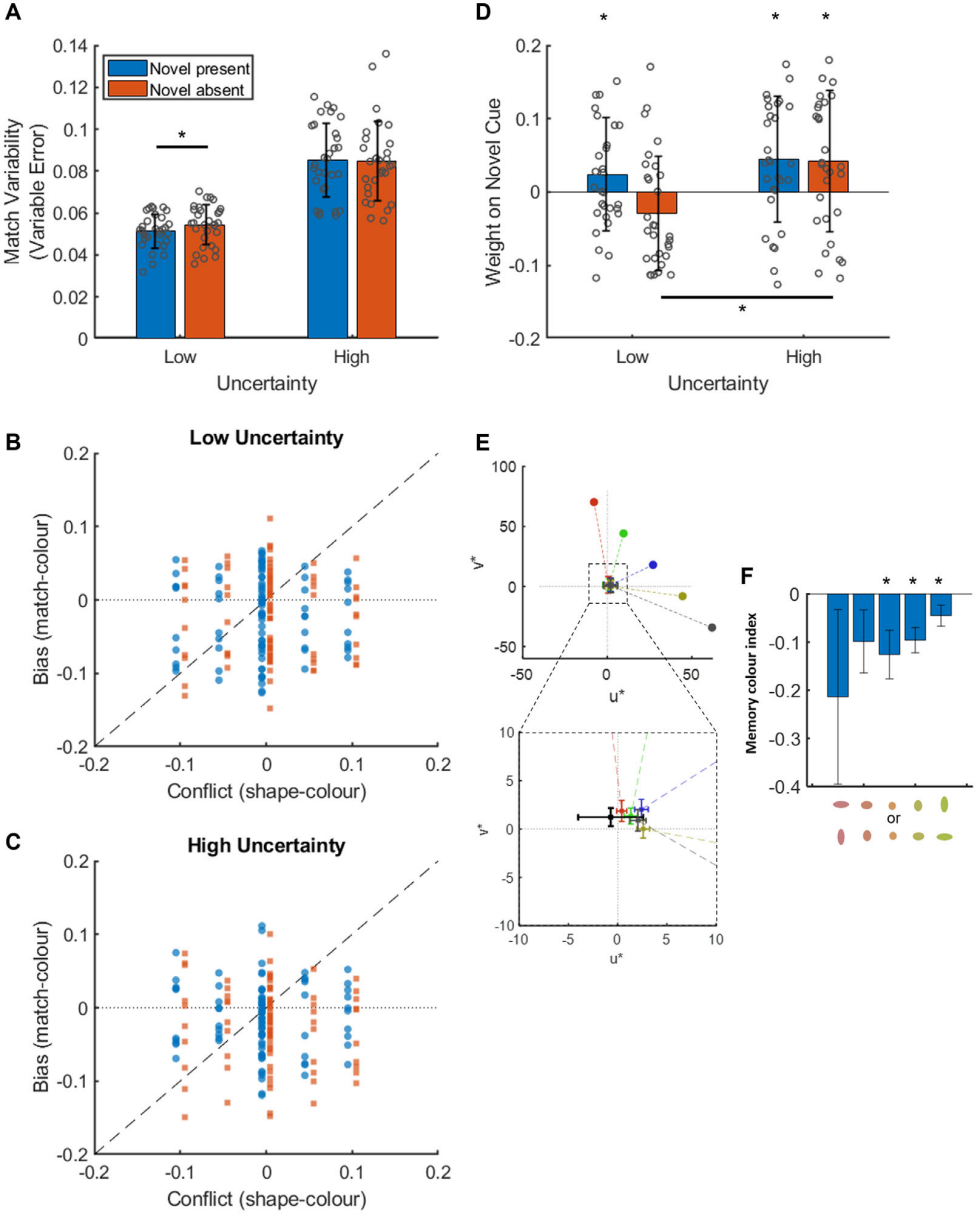
Results from Experiment 2. (A) Matching variability (variable error) for each level of uncertainty split by novel cue presence. (B and C) Individual matching biases (matched color – reference color) for an example participant plotted against the conflict between the shape (novel) and color (familiar) cues in the reference patch (shape cue value – color cue value) in the low (B) and high (C) uncertainty conditions. Blue dots are when the novel cue was present in the matching patch. Orange squares are when the novel cue was absent from the matching patch. (D) The inferred weight on the novel cue in each condition. (E) Example raw color matches from the fourth set and the calculated memory color indices. The black cross represents the average control match. Error bars are ±1 standard deviation.

As we did for Experiment 1, in Figures 6B and 6C we show data from an example participant on trials where the novel shape cue conflicted with familiar cues to color. Much like in Experiment 1, these figures suggest that conflicting novel shape cues have little effect on the bias in color matches. However, averaging across all participants (Figure 6D), there was a small but significant positive weight on the novel cue in all conditions except when uncertainty was low and the novel cue was absent from the matching patch: novel present, low uncertainty: $\bar{x}\;=\;0.02,\;\;t\left(29\right)\;=\;1.7,\;\;p\;=\;\;.05$; novel absent, low uncertainty: $\bar{x}\;=\;-0.03,\;t\left(29\right)\;=\;-2.06,\;p\;=\;0.976$; novel present, high uncertainty: $\bar{x}\;=\;0.04,\;\;t\left(29\right)\;=\;2.87,\;\;p\;=\;0.004$; novel absent, high uncertainty: $\bar{x\;}=\;0.04,\;t\left(29\right)\;=\;2.37,\;p\;=\;0.012$. Under the assumption of reliability-weighted averaging, we expected the weight on the novel cue to increase with increasing uncertainty. This was true when the novel cue was absent from the matching patch, significantly greater weight in the high condition, *t*(29)  =  3.79,  *p*  <  0.001 (compare orange bars in Figure 6D), but not when the novel cue was present in the matching patch, *t*(29)  = 0.97,  *p*  =  0.171 (compare blue bars in Figure 6D).

#### There was no evidence of a memory color effect

As an example, Figure 6E shows the mean achromatic match for each color tested in the fourth and final set of achromatic matches. The inset enlarges the area immediately surrounding the matches where the control match (black error bars) can also be seen. It is clear from this figure that all matches are shifted away from the control match. To quantify this effect, we calculated memory color indices (see the Data analysis section). In a 5 (color) × 4 (match set) repeated measures analysis of variance there was no interaction effect of match set and color, *F*(12, 336)  =  0.96,  *p*  = 0.486, and no main effect of match set, *F*(3, 84)  =  0.86,  *p*  =  0.466, so we collapsed memory color indices over matching sets to get a mean memory color index for each set of matches (Figure 6F). The mean memory color index was consistently less than zero for all five colors tested but only significantly so for three of them, 0.1: *m*  =   −0.21,  *t*(28)  =   −1.18,  *p*  = 0.124; 0.3: *m*  =   −0.1,  *t*(28)  =   −1.51,  *p*  =  0.071; 0.5: *m*  =   −0.13,  *t*(28)  =   −2.49,  *p*  = 0.009; 0.7: *m*  =   −0.1,  *t*(28)  =   −3.67,  *p*  < 0.001; 0.9: *m*  =   −0.05,  *t*(28)  =   −2.06,  *p*  =  0.024. In other words, there was a significant effect in the opposite direction of a classic memory color effect for three of the colors tested.

## General discussion

We extend the limited work on efficient use of novel cues from the domain of object localization (judging where targets are) to object matching or recognition (judging what they are). We tested participants in a short-term training study with a novel shape–color association, making object shape a useful cue to object color within the task. We tested for two signatures of reliability-weighted averaging of the novel shape cue with familiar cues to color (a variability reduction and cue reweighting) and for a perceptual effect of the novel cue. We found signatures of reliability-weighted averaging of the novel shape cue and familiar cues to color in both experiments we presented. Participants showed a reduction in color matching variability in both experiments and cue reweighting with changes in uncertainty in Experiment 2. However, our results overall suggest that the ability to quickly learn novel cues and take a reliability-weighted average of novel and familiar cues is not immediately accompanied by common perceptual effects.

In Experiment 1, we found weak evidence for reliability-weighted averaging. There was a variability decrease when the novel cue was present in the matching patch, but only in the low uncertainty condition, and the weight placed on the novel cue was not significantly different from zero in any condition. We also found mixed evidence of a perceptual effect of the novel shape cue. In simultaneous matches, matching variability was lower when the novel cue was present in the matching patch, which we interpret as evidence for a perceptual effect of the novel cue, because there was a decrease in matching variability when the novel shape cue was present in the matching patch but not when it was absent, even though the same information is available in the reference patch when the novel cue is absent from the matching patch. However, there was no evidence of a perceptual effect from the forced fusion task.

We proposed that the level of mismatch between cue reliabilities in Experiment 1 could have hindered our ability to detect the effects we were looking for and rectified this in Experiment 2 by making familiar cues to color more uncertain and better matched in reliability to the novel shape cue to color. As intended, this manipulation encouraged participants to place more weight on the novel shape cue in Experiment 2, where the weight placed on the novel cue was significantly greater than zero in three of four conditions. However, there was only a significant increase in the weight on the novel cue with increased familiar cue uncertainty when the novel cue was absent from the matching patch. We also tested for a perceptual effect of the novel cue in Experiment 2 using a method that did not require well-matched cue reliabilities by testing for a memory color effect. For three of the five colors tested, we found a significant effect in the opposing direction to the classic memory color effect.

Our results add to the growing literature showing that novel cues can be learned and integrated with familiar cues to enhance perception (Aston et al., 2022; Ernst, 2007; Gibo et al., 2017; Negen et al., 2018, 2021) and extend this finding to the domain of object recognition. We have expanded upon these previous findings by testing for a perceptual effect of the novel cue to color that we introduced. In Experiment 1, we found some evidence of a perceptual effect, as matching variability was lower when the novel cue was present in the matching patch, but no evidence of forced fusion. In Experiment 2, we found a significant memory color effect for three of four colors tested, but it was in the opposite direction to a classic memory color effect.

An inability to acquire memory color effects from novel shape-color associations has been reported elsewhere. In a book chapter, Witzel and Hansen (2015) summarized some of their own unpublished work and the work of others (Huebner and Giesel) obtained through personal communication. In these works, participants were taught novel shape-color associations and the experimenters tested for a memory color effect. Witzel and Hansen (2015) reported that neither study found any evidence of an acquired memory color effect. They did not report how long participants were trained with the novel associations, but it is likely that the exposure periods were short, as in our experiment, and that longer training periods are required before shape becomes diagnostic of an object's color and induces a memory color effect. In unpublished work, Karl Gegenfurtner and colleagues (personal communication, August 2021) trained new color–shape associations (red triangles and blue squares) for at least 10 hours in one laboratory-based study and gave people woolen balls to take home and carry around with them for a few weeks in another study. They also found no evidence of a memory color effect in either study. It is possible that only certain object colors lead to a memory color effect. Witzel and Hansen (2015) hypothesized that memory color effects are strongest for objects with diagnostic colors falling on the daylight axis (the axis that maps out changes in daylight chromaticities across the day—blue to yellow) because achromatic adjustments are more uncertain along this axis. Indeed, Olkkonen et al. (2008) found the largest memory color effect for a banana (yellow) and the smallest memory color effect for a strawberry (red). The former is a color that lies on the daylight axis, whereas the latter lies on a line perpendicular to the daylight axis in the CIELUV uniform color space. Because our colors varied from pinkish to greenish, they may not be the optimal colors to use for inducing a memory color effect. Future studies may find different results using a blue–yellow color gradient.

Why, though, did we observer the opposite of a memory color effect? A simple interpretation is that participants in our experiment did something different to what was asked of them and matched the patches to the associated color rather than gray. However, that seems unlikely given the size of the memory color indices, the matches were only slightly (but consistently) pulled toward the associated color. Another possibility is that adaptation played a role. All shapes seen in our experiment came from a color gradient that was defined as a chord along a hue circle in CIELUV. It is possible that participants adapted to the gradient of colors so that they could not see the slight color left in their gray matches. However, both suggestions are very speculative and further work is needed to explore the opposite effects.

It is possible that we did not see any evidence for the familiar perceptual effects we tested for in our experiments as our participants were only exposed to the novel shape–color association for a short time. Other authors have suggested that the reason children do not combine multiple familiar cues is because cues are still being cross-calibrated throughout development, and that cues are not combined until cross-calibration is complete to retain access to individual cues (Gori, Del Viva, Sandini, & Burr, 2008). A similar explanation could account for participants showing only weak evidence of combination in the experiments presented here. Future experiments should explore the effect of longer-term training. It also remains to be seen to what extent learning to combine new shape and color cues, related to the problem of object recognition (what)—has similar or different perceptual effects to learning to combine other cues, or those related to the problem of object localization (where).

A further limitation of this study is that the shape of the matching patch was fixed in the adjustment trials. Subsequently, even when the novel cue was present in the matching patch, the color and shape did not change simultaneously. However, the shape of the matching patch was the same as that of the reference patch, so we do not expect that it influenced the matched color in a way that would specifically bias the match. There is a possibility that this could have disrupted the learned association, as could the conflict trials. However, we tried to minimize this disruption with the top-up exposure blocks and by interleaving no-conflict trials throughout the experiment.

The study was also limited by the options that were available to us for removing the shape cue and creating the novel absent conditions. We chose to do this by showing a square stimulus rather than an oval with varying axis ratio when the novel cue was absent. However, this factor did not necessarily remove the novel cue, as there was still a shape present and observers could have made an assumption about what that shape corresponded with.

## Conclusions

In two experiments observers showed signatures of reliability-weighted averaging of a newly learned novel shape cue to color with familiar cues. Specifically, they showed a precision-improvement in both experiments and reweighting of the newly learned shape cue with changes in uncertainty in Experiment 2. However, we find no evidence that reliability-weighted averaging of novel and familiar cues is accompanied by perceptual effects, finding no evidence for forced fusion in Experiment 1 and the opposite of a memory color effect in Experiment 2. These results suggest that, although people rapidly learn new statistical regularities to optimize their perceptual decisions, these are not immediately (with short training) accompanied by familiar perceptual effects. How much experience is needed to see familiar perceptual effects with newly learned cues is an open question for future research.
